# Residential exposure to deadly gun violence and accelerated biological aging in a national sample of U.S. adolescents

**DOI:** 10.1016/j.ssmph.2026.101937

**Published:** 2026-06-19

**Authors:** Connor D. Martz, Mateo P. Farina, Julia M. Fleckman, Katherine P. Theall, Rayna Gasik, Lexie M. Contreras, Ethan Smith, Colter Mitchell, Lauren Gaydosh

**Affiliations:** a Department of Population Health Sciences, University of Central Florida College of Medicine, 6850 Lake Nona Blvd, Orlando, FL, 32827, USA; b Department of Human Development and Family Science, University of Texas at Austin, 108 E. Dean Keeton St, Stop A2702, Austin, TX, 78712, USA; c Population Research Center, University of Texas at Austin, 305 E. 23rd St, Stop G1800, Austin, TX, 78712, USA; d Washington University in St. Louis School of Public Health, 660 S. Euclid Ave, St. Louis, MO, 63110, USA; e Celia Scott Weatherhead School of Public Health and Tropical Medicine, Tulane University, 1440 Canal St, New Orleans, LA, 70112, USA; f Violence Prevention Institute, Tulane University, 1440 Canal St, New Orleans, LA, 70112, USA; g Institute for Social Research, University of Michigan, 426 Thompson St, Ann Arbor, MI, 48104, USA; h Department of Sociology, University of North Carolina at Chapel Hill, 155 Hamilton Hall, CB #3210, Chapel Hill, NC, 27599, USA; i Carolina Population Center, University of North Carolina at Chapel Hill, 123 W. Franklin St, Chapel Hill, NC, 27516, USA

**Keywords:** Gun violence, Biological aging, DNA methylation, Epigenetic, Life course

## Abstract

**Background:**

Gun violence is a traumatic and pervasive neighborhood stressor with far-reaching consequences for population health. However, less is known about how gun violence exposure becomes biologically embedded during adolescence, a sensitive period for shaping aging trajectories. We examined whether residential exposure to deadly gun violence is associated with accelerated biological aging in a diverse sample of U.S. adolescents.

**Methods:**

The sample included 1,781 15-year-olds in the Future of Families and Child Well-being Study (2014-2017). Neighborhood-level gun violence was defined as the count of fatal shootings within 1600 m (∼1 mile) of respondents’ homes in the prior year. Accelerated biological aging was measured using a composite of three epigenetic clocks (GrimAge, PhenoAge, DunedinPACE). Propensity score matching balanced exposed and unexposed adolescents on race/ethnicity, socioeconomic characteristics, and area-level crime; weighted linear regression models targeting the average treatment effect on the treated then estimated associations between exposure counts and biological aging in the matched sample (n = 499).

**Results:**

Approximately half of adolescents (51.3%) lived near at least one deadly shooting in the prior year. Among exposed adolescents in the matched sample (n = 265), the mean exposure count was 3.24 (SD = 3.50). Greater exposure to deadly gun violence was associated with accelerated biological aging (β = 0.033; 95% CI, 0.003 to 0.064).

**Conclusions:**

Adolescent exposure to deadly gun violence may be linked to epigenetic markers of aging-related health risk, suggesting that chronic early-life exposure may have lasting implications for long-term health and aging.

## Highlights

• Over 50% of adolescents were exposed to nearby incidents of deadly gun violence in the prior year.
• Black and Hispanic adolescents faced greater likelihood and intensity of exposure.
• Greater exposure count was associated with epigenetic indicators of accelerated biological aging.
• Exposed adolescents showed estimated differences of ∼2.5 months in biological age acceleration and ∼5.3% faster pace of aging.
• Early-life exposure to gun violence may increase risk of aging-related chronic disease.

## 1 Introduction

The United States faces an epidemic of gun violence with far-reaching and unequally distributed consequences for population health (Kravitz-Wirtz et al., 2022; US Surgeon General, 2024). Beyond lives lost and nonfatal injuries, neighborhood gun violence represents a chronic source of contextual stress that directly and indirectly influences how individuals navigate daily life (Sharkey, 2018). Exposure to gun violence is often conceptualized as an acute traumatic event, such as direct victimization or witnessing a shooting. However, for many residents of high-violence neighborhoods, gun violence is not experienced as a singular incident but as a persistent environmental threat (Sharkey, 2018). The ongoing possibility of nearby shootings, the sound of gunfire, memorials marking loss, and heightened vigilance in daily routines constitutes a socially embedded and reoccurring stressor (Bancalari et al., 2022; Hardiman et al., 2019; Opara et al., 2020; Tam et al., 2024). Thus, residence in violent neighborhoods may harm well-being not only through discrete violent encounters, but also through sustained exposure to environments in which the threat of violence is chronically elevated and pervasive (Riehm et al., 2021; Semenza & Kravitz-Wirtz, 2024; Sharkey, 2018).

Exposure to such chronically threatening environments may be particularly consequential during adolescence—a sensitive period of rapid growth, neurobiological maturation, and epigenetic changes that recalibrate stress response systems across the transition to adulthood (Eiland & Romeo, 2013; Semenza & Kravitz-Wirtz, 2024; Turner et al., 2019). During this stage, social contexts play a critical role in shaping regulatory, emotional, and physiological functioning in ways that influence lifelong health trajectories. Growing evidence links early-life exposure to gun violence with adverse mental health and developmental outcomes, including greater risk of suicidality, depression, anxiety, posttraumatic symptoms, substance use, and neurobehavioral impairments (Abba-Aji et al., 2024; Bancalari et al., 2022; Holloway et al., 2023). Early-life exposure has also been associated with increased risk of chronic physical conditions such as asthma and hypertension (Ford & Browning, 2014; Ramratnam et al., 2015). Yet, despite accumulating evidence of these downstream outcomes, we have limited understanding of how early-life exposure to gun violence becomes biologically embedded to shape physical health risks throughout the life course (Semenza & Kravitz-Wirtz, 2024).

Social epidemiological frameworks posit that sustained exposure to environmental threats such as gun violence can increase biological risks through stress-mediated pathways (Krieger, 2001; Semenza & Kravitz-Wirtz, 2024). Repeated activation of stress response systems in the context of persistent vigilance and threat can disrupt neuroendocrine, inflammatory, and immune processes. Consistent with this framework, exposure to neighborhood violence—regardless of firearm involvement—has been associated with elevated inflammation, hormonal dysregulation, altered immune function, and impaired corticolimbic connectivity among youth (Chat et al., 2022; Finegood et al., 2020; Gard et al., 2022; Gresham et al., 2025; Miller et al., 2022). Chronic violence exposure can prolong heightened physiological states which accumulate “wear and tear” over time. During adolescence, these processes may result in deviations from typical developmental timing and contribute to the acceleration of age-related health declines (López-Otín et al., 2013). Accelerated biological aging occurs when an individual's biological age, as estimated by various markers of physiological deterioration, exceeds their chronological age (Horvath & Raj, 2018). Growing research has linked accelerated biological aging to increased risk of age-related disease in older adulthood, suggesting that acceleration early in the life course may have adverse health consequences decades later (Danese & McEwen, 2012).

Biological age can be measured using epigenetic “clock” biomarkers that estimate how slowly or rapidly an individual is aging at a molecular level based on changes in specific CpG sites (cytosine-guanine dinucleotide pairs where DNA methylation occurs). Unlike individual stress markers that fluctuate over short time scales, epigenetic clocks capture accumulated biological risks derived from DNA methylation (DNAm) patterns associated with morbidity, disability, and mortality in later life (Horvath & Raj, 2018; Mitchell et al., 2016; Ryan, 2021). Importantly, epigenetic clocks are sensitive to chronic stress and early-life social adversity, including socioeconomic disadvantage and trauma (Chang et al., 2024; Del Toro et al., 2024; Harris et al., 2024; Martz et al., 2024; Musci et al., 2023). They have also been linked to adverse pediatric outcomes that are frequently associated with exposure to gun violence, such as psychopathology, obesity, and impaired cognition. (Aikins et al., 2025; Etzel et al., 2022; Raffington et al., 2023). Despite the promise of epigenetic-based aging measures for detecting early biological embedding of social adversity long before the onset of clinical symptoms, their relationship with gun violence exposure during adolescence remains unexplored (Jackson et al., 2023; Mitchell et al., 2016).

The current study addresses this gap and responds to calls for integrating epigenetic aging biomarkers with rich contextual measures in life course research (Mitchell et al., 2016). We examine whether residential exposure to deadly gun violence is associated with accelerated biological aging among a large, racially and socioeconomically diverse sample of U.S. adolescents from the Future of Families and Child Wellbeing Study (FFCWS; 2014-2017) (Reichman et al., 2001). We tested the hypothesis that greater residential exposure to deadly gun violence within 1600 m of respondents’ homes in the prior year is associated with epigenetic-based age acceleration at age 15. Building on prior FFCWS research (Aubel et al., 2023; Bruns et al., 2025; Buggs et al., 2022; Gard et al., 2022; James et al., 2021; Kravitz-Wirtz et al., 2022; Leibbrand et al., 2020, 2021; Zhang et al., 2025), this study contributes novel evidence on the biological pathways through which neighborhood gun violence may shape age-related health outcomes across the life course.

## 2 Methods

### 2.1 Participants

The Future of Families and Child Wellbeing Study (FFCWS) is a racially and socioeconomically diverse birth cohort of 4,898 youth born across 20 US cities from 1998 to 2000, oversampled for births in single-headed households (Reichman et al., 2001). Follow-up assessments occurred at Years 1, 3, 5, 9, and 15, corresponding to participants' chronological age at each assessment. A subgroup of 1,981 participants was enrolled in the biomarker sub-study, which assayed DNAm from saliva samples collected at Year 9 and Year 15 (Bendheim-Thoman Center for Research on Child Wellbeing, 2023). Enrolment in the biomarker sub-study was based on completion of in-home visit activities at Year 9 and provision of saliva sample at Year 15. Inclusion criteria for the current study included complete data at both Year 9 and Year 15 on DNAm, deadly gun violence, and all covariates, resulting in an overall analytic sample of 1,781 adolescents. The Princeton University institutional review board granted ethical approval. Informed written consent was obtained from participants’ legal guardians.

### 2.2 Measures

#### 2.2.1 Epigenetic-based biological aging

We used factor analyses to create composite summary scores of biological aging at Year 9 and Year 15 derived from three epigenetic clocks assayed from saliva samples and predictive of age-related morbidity and mortality (GrimAge, PhenoAge) and pace of aging (DunedinPACE) (Belsky et al., 2022; Bendheim-Thoman Center for Research on; Child Wellbeing et al., 2023; Levine et al., 2018; Lu et al., 2019). Factor loadings are presented in Table S1; scalar measurement invariance was supported across both time points. For GrimAge and PhenoAge, we operationalized accelerated aging as the standardized residuals of principal component (PC) versions of each clock regressed on chronological age. DunedinPACE inherently measures the rate of change in biological age relative to one year of chronological age. We selected these clocks because they reflect saliva-derived indicators of disease-relevant physiological risk as opposed to clocks developed for pediatric or buccal tissue (e.g., PedBE) which prioritize chronological-age prediction (in sensitivity analyses, PedBE estimates were directionally consistent with our primary findings) (McEwen et al., 2020). Additional information on the collection, processing, and operationalization of the outcome measure is presented in the Supplemental Methods.

#### 2.2.2 Exposure to deadly gun violence

Adolescents’ residential exposure to deadly gun violence was measured as the count of incidents (excluding suicides) that occurred in the past year within 1,600 m (approximately one mile) of their Year 15 residential addresses (Bendheim-Thoman Center for Research on Child Wellbeing & Columbia Population Research Center, 2019b, Bendheim-Thoman Center for Research on Child Wellbeing & Columbia Population Research Center, 2019a). Geocoded data on incidents of deadly gun violence between January 1, 2014 and October 5, 2017 were obtained from the Gun Violence Archive, an independent research group that aggregates incident data through automated queries and manual searches of media, government, and law enforcement sources (Gun Violence Archive, 2023). Approximately 76% of the sample completed Year 15 data collection at least one year after the Gun Violence Archive start date (Bendheim-Thoman Center for Research on Child Wellbeing & Columbia Population Research Center, 2019a). A buffer of 1,600 m was selected as the primary exposure radius as it represents the maximum distance available in the FFCWS dataset and is consistent with prior research using this dataset characterizing residential gun violence exposure across a range of spatial buffers (Aubel et al., 2023; Buggs et al., 2022; Kravitz-Wirtz et al., 2022; Leibbrand et al., 2020). Sensitivity analyses at 1,000m and 500m buffers are reported to assess spatial robustness.

#### 2.2.3 Covariates

Analyses adjusted for the following covariates: chronological age, sex, self-identified race/ethnicity, maternal income-to-poverty ratio, maternal educational attainment (< high school, high school, some college, college), census tract percent poverty, and county-level violent crime rates from Uniform Crime Reports. Consistent with the study design, which oversampled births to unmarried mothers, socioeconomic covariates reflect maternal characteristics, which are available for the full analytic sample and are standard in prior FFCWS-based research (e.g., Gard et al., 2022; Kravitz-Wirtz et al., 2022; Leibbrand et al., 2020).

We also adjusted for technical covariates related to epigenetic clocks, including maternal prenatal smoking, DNAm array chip (EPIC, 450K), and cell type estimates derived by FFCWS via reference-based deconvolution (abundance of plasmablasts, CD8^+^ CD28^−^ CD45RA- T cells, and naïve CD8 T cells), with full procedural details located in the Supplement and FFCWS documentation (Bendheim-Thoman Center for Research on Child Wellbeing & Columbia Population Research Center, 2019b; Horvath, 2013). To account for prior biological aging, Year 9 epigenetic clock summary score values were included as a covariate in final models.

### 2.3 Statistical analyses

Analyses were conducted from June to November 2025 using R version 4.2.2 (R Project for Statistical Computing). Regression estimates are presented as standardized coefficients (SD units) with 95% confidence intervals based on heteroskedasticity-robust standard errors (HC1) in full sample models and cluster-robust standard errors (clustered by matched pair) in propensity score-matched models; p-values are reported for moderation tests and sample characteristic comparisons. Minimal geographic clustering of participants did not warrant the use of hierarchal modeling approaches. No adjustment for multiple comparisons was applied, as supplemental analyses were conducted to triangulate and assess the robustness of primary findings rather than to test independent hypotheses.

We estimated the association between incident counts within 1,600m and biological aging following methodological recommendations for studying the long reach of violence (Sharkey, 2018). This approach conceptualizes exposure to gun violence not merely as an individual-level risk factor but as a feature of the residential context itself. Acknowledging the underlying structural forces that generate racial disparities in exposure, we first examined evidence of residual sorting in the overall sample using stepwise regression models. Model 1 adjusted for outcome-relevant covariates of chronological age, sex, maternal prenatal smoking, array chip, and cell type estimates. Model 2 further adjusted for maternal income-poverty ratio and maternal education; Model 3 further adjusted for census tract percent poverty; and Model 4 further adjusted for self-identified race/ethnicity. The final stepwise model in the overall sample (Model 5) further adjusted for prior values of time-varying covariates at Year 9, including biological age acceleration and county-level violent crime rates.

To account for potential residential sorting processes, we used a propensity score approach to identify a matched sample for our primary analysis. Compared to alternative weighting approaches for continuous exposures, propensity score matching is more conceptually aligned with our target estimand of the average treatment effect on the treated (ATT). Matching on the treated better isolates the contextual threat distinguishing violent from otherwise similar non-violent environments, consistent with conceptualizing gun violence as a spatially embedded condition (Sharkey, 2018). Alternative methods for continuous exposure propensity scores, which overlook this key conceptual premise, resulted in a smaller effective sample size and worse covariate balance.

We first estimated the probability of exposure to at least one incident of deadly gun violence within 1,600 m of adolescents’ homes to establish a comparable sample of treated (any exposure) and control (no exposure) groups. Treated participants were matched with unexposed participants of the same race/ethnicity based on comparable individual- and census tract-level socioeconomic characteristics at Year 9 and Year 15, including prior area-level exposure to gun violence (Year 9 county-level violent crime rates). Because census tract poverty and race/ethnicity are constitutive of the structural processes that generate differential residential exposure to gun violence – rather than individual-level confounders in the conventional sense – fully adjusting for these factors in unmatched regression models risks overadjustment bias (Bailey et al., 2017; Schisterman et al., 2009; VanderWeele & Robinson, 2014; Westreich & Greenland, 2013). Our propensity score-matched design addresses this by balancing structural characteristics across exposed and unexposed adolescents prior to estimation, targeting a within-stratum contextual comparison. After ensuring covariate balance and overlapping distributions, we used weighted regression to estimate the association between the continuous exposure count and biological aging. This approach aligns with recommendations to capture exposure intensity. Additional details on propensity score matching analyses are provided in the Supplementary Methods.

Our primary results reflect weighted regression estimates targeting the ATT in the matched sample, adjusted for non-matching, outcome-related covariates of chronological age, sex, prior (Year 9) biological aging, maternal prenatal smoking, cell type estimates, and array chip. We conducted multiple sensitivity analyses to assess the robustness of main findings, including models that estimated associations at levels of exposure intensity and continuous exposure counts at 1,000m and 500m. We also fit models accounting for concurrent county-level violent crime rates at Year 15 (as both a matching parameter and covariate in weighted regression models) and residential mobility between Years 9 and 15. Finally, we compared estimates using the biological aging summary score with those for each epigenetic clock. R code is available at our GitHub repository. https://anonymous.4open.science/r/gunviolence_biologicalaging-8DD6/https://anonymous.4open.science/r/gunviolence_biologicalaging-8DD6/.

## 3 Results

### 3.1 Sample characteristics

The full analytic cohort included 1,781 adolescents with a mean [SD] age of 15.53 [0.63] years (Table S2). Approximately half of adolescents (51.32%) lived within 1,600m of at least one fatal shooting in the prior year (mean [SD] incident count, 2.38 [4.48]; range, 0-44). Exposure to deadly gun violence varied by self-identified race and ethnicity (*p* < 0.001). Black and Hispanic adolescents were most likely to live in neighborhoods affected by deadly gun violence, and they also faced the greatest exposure intensity (all *p* < 0.001).

We identified a matched sample of 499 adolescents by creating two groups (treatment n = 265, control n = 234)^1^ that differed on exposure status but were similar (all standardized mean differences <0.10) on all individual- and tract-level covariates (Table 1; Table S2; Figs. S1–S2). The mean exposure count among the matched treatment group was 3.24 (SD = 3.50) incidents (Table 1). Sociodemographic characteristics of the matched sample closely resembled those of the full analytic cohort (Table S2).

**Table 1:** Weighted descriptive statistics for the matched sample of adolescents from Year 15 in the Future of Families and Child Well-being Study.

Variable	Matched (N = 499)	Control (n = 234)^a^	Treatment (n = 265)	Std. Mean Difference
Propensity score (any exposure), mean (SD)	0.56 (0.22)	0.56 (0.22)	0.56 (0.22)	0.03
Exposure count (1600m), mean (SD)	1.72 (3.02)	0.00 (0.00)	3.24 (3.50)	0.86
Biological aging (std.), mean (SD)	0.14 (1.09)	0.14 (1.18)	0.15 (1.00)	0.01
Chronological age, mean (SD), years	15.60 (0.69)	15.59 (0.67)	15.61 (0.72)	0.04
Sex, No. (%)				
Female	270 (54.05)	130 (55.44)	140 (52.83)	−0.03
Male	229 (45.95)	104 (44.56)	125 (47.17)	−0.04
Self-identified race/ethnicity, No. (%)				
Non-Hispanic Black	288 (57.74)	135 (57.74)	153 (57.74)	0.00
Hispanic	160 (32.08)	75 (32.08)	85 (32.08)	0.00
Non-Hispanic White	38 (7.55)	18 (7.55)	20 (7.55)	0.00
Other/Multiple Race	13 (2.64)	6 (2.64)	7 (2.64)	0.00
Maternal educational attainment, No. (%)				
Less than high school	117 (23.38)	50 (21.23)	67 (25.28)	0.04
High school	90 (18.08)	43 (18.46)	47 (17.74)	−0.01
Some college	228 (45.62)	102 (43.43)	126 (47.55)	0.04
College	65 (12.93)	40 (16.89)	25 (9.43)	−0.07
Maternal income-to-poverty ratio, mean (SD)	1.49 (1.41)	1.50 (1.41)	1.48 (1.41)	−0.00
Census tract poverty, mean (SD), percent	23.29 (11.88)	23.25 (12.02)	23.32 (11.76)	0.01
Y9 County Violent Crime Rate, mean (SD)	690.61 (307.36)	683.27 (313.68)	697.10 (301.51)	0.04

a Effective sample size (ESS) = 153.61.

### 3.2 Associations between gun violence exposure and biological aging in the overall sample

We first conducted stepwise regression models in the overall sample (n = 1,781) to examine confounding and assess evidence of residential sorting processes. Standardized estimates from Model 1 indicated that greater prior-year exposure to deadly gun violence within 1,600 m of home was associated with accelerated biological aging (β = 0.060, 95% CI, 0.037 to 0.083) after adjusting for chronological age, sex, maternal prenatal smoking, array chip, and cell types. This association persisted after adjusting for individual-level socioeconomic factors (Model 2: β = 0.049, 95% CI, 0.025 to 0.072). Further adjustment for census-tract poverty (Model 3: β = 0.025, 95% CI, −0.000 to 0.050) and self-identified race/ethnicity (Model 4: β = 0.006, 95% CI, −0.018 to 0.031) attenuated estimates and revealed confidence intervals spanning zero. Similar associations were observed after adjusting for time-varying covariates and prior biological aging at Year 9 (Model 5: β = 0.005, 95% CI, −0.011 to 0.020). The attenuation of estimates across stepwise models, particularly after adjusting for tract poverty and race/ethnicity, is consistent with the role of structural factors and residential sorting processes that shape both exposure to neighborhood gun violence and accelerated biological aging.

### 3.3 Average treatment effect on the treated (ATT) in matched sample

**Fig. 1:**
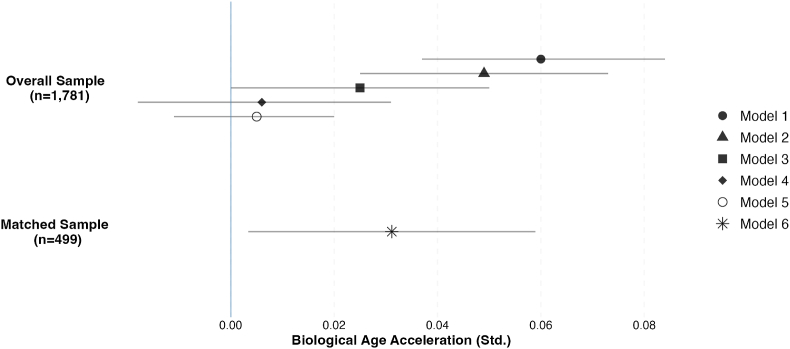
Associations between exposure to gun violence and biological age acceleration in the overall (n = 1781) and matched sample (n = 499) of adolescents in the Future of Families and Child Wellbeing Study (2014-2017). Plotted estimates for the overall sample are from stepwise regression analyses. Model 1 adjusted for Y15 covariates of chronological age, sex, maternal prenatal smoking, array chip, and cell types. Model 2 further adjusted for maternal income-to-poverty ratio and maternal educational attainment. Model 3 for census tract percent poverty, Model 4 for self-identified race/ethnicity, and Model 5 for Y9 county-level violent crime rate and time-varying Y9 covariates, including prior biological aging, cell type estimates, chronological age, maternal income-to-poverty ratio, census tract percent poverty. The primary analysis (Model 6) reflects main estimates from fully adjusted weighted regression models in the matched sample targeting the average treatment effect on the treated (N = 499; treatment n = 265, control n [ESS] = 234 [153.61]).

We next estimated our primary model, targeting the ATT using weighted regression in a propensity score-matched sample (n = 499; Table S3). Among adolescents living in neighborhoods affected by deadly gun violence, greater exposure was associated with accelerated biological aging (Model 6: β = 0.033; 95% CI, 0.003 to 0.064), relative to the unexposed counterfactual. This estimate was similar in magnitude to the Model 3 estimate from the overall sample after adjustment for tract poverty (Fig. 1). Exploratory moderation analyses did not find that estimates from the matched sample differed by race/ethnicity, F(3,479) = 0.565, *p* = 0.638.

### 3.4 Sensitivity analyses

Across sensitivity analyses that tested alternative exposure operationalizations, we observed larger estimates at higher exposure intensities (Fig. S3) and closer residential proximities (Fig. S4). The point estimate for any-versus-none exposure (β = 0.042; 95% CI, −0.025 to 0.108) was also consistent in direction and slightly larger in magnitude compared with primary estimates based on continuous incident counts in Model 6. However, across alternative exposure measures, limited variance and reduced power resulted in wider confidence intervals that overlapped with zero, precluding strong inferences for potential dose-response and spatial sensitivity. Taken together, these sensitivity tests yielded patterns broadly consistent with our primary analysis that modeled exposure intensity using continuous incident counts within 1,600 m of adolescents’ homes (Table S4).

Results from additional sensitivity tests also support our primary findings from weighted regression models (Model 6). The association between continuous exposure count within 1,600m and accelerated biological aging persisted after accounting for concurrent exposure to county-level violent crime rates at Year 15—both as a covariate in weighted regression models and a matching parameter in propensity score analyses. Our primary analysis was also robust to potential exposure misclassification due to data coverage limitations in the Gun Violence Archive: excluding participants with less than one year of exposure data revealed larger estimates (β = 0.051; 95% CI, 0.015 to 0.088) compared to our primary analysis. Accounting for residential mobility between Years 9 and 15 did not alter the results of primary analyses. Weighted regression estimates based on the composite measure of biological aging (Model 6) were consistent across individual epigenetic clocks in both the overall and matched samples (Fig. S5).

## 4 Discussion

This study investigated whether residential exposure to deadly gun violence is associated with epigenetic aging in a diverse, nationwide sample of U.S. adolescents. We found that greater exposure to deadly gun violence within 1,600 m of adolescents’ homes was associated with accelerated biological aging after accounting for structural sorting through propensity score matching. Translating these estimates to an interpretable scale, a 15-year-old living in an urban neighborhood with average levels of deadly gun violence would be estimated as biologically about 2.5 months older according to GrimAge and PhenoAge and aging approximately 5.3% faster according to DunedinPACE compared to unexposed peers.^2^ It is expected that even small deviations between chronological age and biological age, particularly when observed early in the life course, compound over time and may contribute to earlier onset of age-related disease and reduced healthspan (Horvath & Raj, 2018; Mitchell et al., 2016; Raffington, 2024). These associations were consistent across multiple sensitivity analyses examining alternative exposure specifications and adjustment for broader area-level violent crime, although they attenuated in unmatched models with full adjustment for structural covariates.

By leveraging epigenetic clocks, our study provides evidence that early-life exposure to gun violence may influence aging-related biological processes well before the onset of clinical disease. Epigenetic clocks capture accumulated biological risk and are sensitive to chronic stress and adverse social environments (Horvath & Raj, 2018; Musci et al., 2023). Recent evidence suggests that much of their predictive signal reflects immune and inflammatory processes, providing a plausible pathway through which exposure to gun violence may become biologically embedded (Klopack & Crimmins, 2026). The magnitude of the associations observed in our adolescent sample is comparable to those reported for accelerated biological aging following bereavement in midlife (Aiello et al., 2024) and older adulthood (Farina et al., 2024). Although epigenetic clocks offer limited insight into specific biological mechanisms, they predict later-life morbidity and mortality and may serve as early indicators of long-term health risk (Mulder et al., 2024; Warner et al., 2024). At the same time, these measures were developed using non-representative training samples, which may limit generalizability (Chan et al., 2023). While emerging evidence suggests that GrimAge, PhenoAge, and DunedinPACE perform similarly across racial and ethnic groups (Kusters & Horvath, 2025; Mendy & Mersha, 2025), future research is needed to address potential portability concerns, including the development of algorithms trained in nationally representative and diverse populations (Chan et al., 2023).

Our findings align with and extend prior research documenting the developmental and biological consequences of early-life exposure to gun violence. Previous analyses from the Future of Families and Child Wellbeing Study have linked residential exposure to deadly gun violence with altered functional connectivity in brain regions involved in emotion regulation and stress responses (Gard et al., 2022). Other studies among youth have associated violence exposure (broadly-measured) with epigenetic changes related to inflammation and immune function (Ajiboye et al., 2025; Dos Santos Oliveira et al., 2023; Jovanovic et al., 2017; Saadatmand et al., 2021; Serpeloni et al., 2020), as well as dysregulated stress reactivity and shorter telomere length—a marker of cellular aging—among children living in neighborhoods with higher rates of violent crime (Theall et al., 2017). In parallel, early-life exposure to gun violence has been linked to increased risk of asthma in childhood (Ramratnam et al., 2015) and hypertension in adulthood (Ford & Browning, 2014). Together, this body of evidence supports the interpretation that chronic exposure to violent environments may become biologically embedded during development, with potential implications for cardiometabolic and aging-related disease risk later in life.

Our analytic approach was designed to account for the structural forces that sort adolescents into neighborhoods with fundamentally different risk profiles. By matching adolescents exposed to at least one deadly shooting with otherwise similar unexposed adolescents, we estimated associations targeting the average treatment effect on the treated and focused on the qualitative distinction between environments where deadly gun violence is absent versus present. This framework reflects the conceptualization of gun violence as a contextual threat embedded within residential environments rather than solely an individual-level exposure. Patterns observed across sensitivity analyses—suggesting larger estimates at higher exposure intensities and closer residential proximity—were consistent with this perspective, although limited variance and reduced power precluded strong inferences.

It is important to interpret these findings within the broader epidemiological context of gun violence in the United States. We examined fatal shootings between 2014 and 2017, a period that began when gun homicide rates were at a 40-year low (Rees et al., 2022). Although rates increased during the pandemic and have since declined, they remain higher than in 2014, underscoring the public health relevance of our findings (Gun Violence Archive, 2025). Consistent with national patterns, exposure to deadly gun violence in our sample was highly stratified by race and ethnicity, with Black adolescents residing in neighborhoods with substantively higher exposure levels compared to their White peers (Lanfear et al., 2023; Semenza et al., 2025). We did not detect significant differences in associations across racial and ethnic groups, which may reflect limited statistical power or suggest that the adverse biological consequences of gun violence extend broadly across groups. Nevertheless, race and ethnicity strongly shape both exposure intensity and the individual and collective resources available to cope with gun violence. As a result, minoritized adolescents living in disadvantaged neighborhoods may face compounded biological risks due to both greater exposure and chronic activation of stress response systems (Miller et al., 2021; Semenza & Kravitz-Wirtz, 2024). Taken together, our estimates may represent a conservative assessment of the biological consequences of contemporary levels of gun violence, particularly in communities experiencing concentrated poverty (Kravitz-Wirtz et al., 2022).

Efforts to reduce the far-reaching health burden of gun violence must address the structural conditions that produce unequal exposure. Community violence is shaped by systemic inequities, including structural racism and chronic disinvestment in predominantly Black and Hispanic communities (Mann et al., 2024; Semenza & Kravitz-Wirtz, 2024). While increased policing has often been deployed to address gun violence, aggressive and disparate policing practices have been shown to undermine perceptions of safety, erode social cohesion, and in some cases exacerbate stress-related health risks (Crifasi et al., 2022; Sampson et al., 1997; Theall et al., 2022). Exposure to over-policing and police violence has itself been linked to accelerated aging (Del Toro et al., 2024; McFarland et al., 2018) and heightened stress biomarkers (Browning et al., 2021). Accordingly, growing evidence indicates that interventions are most effective when they prioritize equitable, place-based approaches that reduce violence without compounding the health toll of chronic stress and marginalization in affected communities (Dong et al., 2024; Rosenberg, 2021; Semenza & Kravitz-Wirtz, 2024).

### 4.1 Limitations

This study is observational, and results should not be interpreted as causal. Exposure was measured based on deadly shootings occurring within 1,600 m of adolescents’ homes in the year prior to age 15 DNAm measurement; we were unable to capture all incident exposures between Year 9 and Year 15 assessments, nor those prior to age 9 or occurring beyond the maximum 1,600 m buffer available in the FFCWS data. Despite these data constraints, prior-year exposure likely reflects enduring features of neighborhood context rather than isolated events, consistent with the geographic persistence of gun violence and the persistence of findings after adjustment for earlier crime rates and residential mobility. We could not assess potentially salient dimensions of exposure, including non-fatal shootings, police-involved killings, direct witnessing of violence, or personal connections to victims (Holloway et al., 2023; Opara et al., 2020; Semenza & Kravitz-Wirtz, 2024). Moreover, the Gun Violence Archive relies heavily on media reports, which may introduce systemic underreporting, particularly for incidents involving Black individuals and men (Gobaud et al., 2023). Together with the lower variance in some exposure measures, these limitations likely biased estimates toward the null and contributed to wider confidence intervals, limiting our ability to draw strong conclusions regarding dose-response and spatial sensitivity. Future research would benefit from distance-weighted exposure measures that better capture spatial gradients in chronic stress burden.

An additional limitation is that the epigenetic clocks used here were developed and validated primarily in blood, whereas the FFCWS assayed DNAm from saliva, a heterogeneous mixture of immune and epithelial cells. Although blood-derived clocks are increasingly applied to salivary DNAm data and have detected meaningful associations with social exposures and health outcomes in adolescent samples (Del Toro et al., 2024; Dos Santos Oliveira et al., 2023; Martz et al., 2024), tissue source can influence absolute clock values and effect size estimates (Apsley et al., 2025). Adjusting for cell type estimates, as done in this study, accounts for the possibility that observed differences in epigenetic age reflect shifts in the cellular makeup of the saliva sample rather than the intrinsic aging-related changes in methylation (Horvath, 2013). We therefore interpret the current epigenetic clocks not as saliva-specific aging measures but as saliva-derived indicators of broader biological aging and physiological risk.

Despite these limitations, our study has several important strengths. We leveraged a large, racially and socioeconomically diverse cohort with epigenetic clocks and spatial exposure to gun violence, linked from one of the only validated national data sources capturing fine-grained geographic coverage. By summarizing multiple epigenetic clocks into a latent composite measure, we captured global aging-related processes while reducing measurement noise. Our theory-driven design explicitly addressed residential sorting processes, and although matching reduced the analytic sample size, effect estimates in the matched sample were consistent with minimally adjusted models in the full cohort. Collectively, these strengths support the methodological rigor of the analysis and reflect a broader shift toward more spatially nuanced, methodologically rigorous, and equity-conscious approaches to studying gun violence and health (Sharkey, 2018).

### 4.2 Conclusions

Using a large and diverse national sample of US adolescents, this study provides evidence consistent with an association between residential exposure to deadly gun violence and accelerated biological aging. These findings underscore the urgency of preventing gun violence and investing in community-based supports and structural interventions that address the root causes of violence. Reducing gun violence may not only prevent injury, death, and bereavement, but also mitigate the potential long-term biological risks of chronic stress, with lasting benefits for population health across the life course.

## CRediT authorship contribution statement

**Connor D. Martz:** Writing – original draft, Formal analysis, Conceptualization. **Mateo P. Farina:** Writing – review & editing, Conceptualization. **Julia M. Fleckman:** Writing – review & editing, Writing – original draft. **Katherine P. Theall:** Writing – review & editing, Methodology, Conceptualization. **Rayna Gasik:** Writing – review & editing. **Lexie M. Contreras:** Writing – review & editing. **Ethan Smith:** Writing – review & editing. **Colter Mitchell:** Writing – review & editing, Funding acquisition, Data curation. **Lauren Gaydosh:** Writing – review & editing, Supervision, Conceptualization.

## Ethical statement

This study used publicly available, deidentified survey and biomarker data collected by the Future of Families and Child Wellbeing Study (FFCWS). Geocoded data on exposure to gun violence were accessed through an approved restricted-use data contract. FFCWS study protocols were approved by the Princeton University IRB Office (#0000005767). Informed written consent was obtained from participants’ legal guardians.

## Funding/support

This study was supported by the National Institute of Child Health and Human Development under award numbers T32HD007081 and P2CHD042849; the National Institute on Aging under award numbers R00AG076964, R01AG071071, R25AG05327, and P30AG066614; the Centers for Disease Control and Prevention under award number U01CE003384; and the National Institute of Justice under award number DOJ 15PNIJ-23-GG-04267-CVIP. Funding for the Future of Families and Child Wellbeing Study was provided by the National Institute of Child Health and Human Development (R01HD036916, R01HD076592, R01HD039135, R01HD040421); the National Institute of Minority Health and Health Disparities (R01MD011716); and the National Institute of Mental Health (R01MH103761). The content is solely the responsibility of the authors and does not necessarily represent the official views of the National Institutes of Health, Centers for Disease Control and Prevention, and National Institute of Justice. Role of study sponsors**:** The funders had no role in the design and conduct of the study; collection, management, analysis, and interpretation of the data; preparation, review, or approval of the manuscript; and decision to submit the manuscript for publication.

## Declaration of interest statement

The authors declare that they have no competing interests.

## Data Availability

Data can be accessed via a restricted-use data contract with the Future of Families and Child Wellbeing Study.

## Footnotes

1 Due to unequal matching weights assigned by the full matching procedure, the effective sample size (ESS) for the control group is 153.61 (Kish 1965); full details are provided in the Supplemental Methods.

2 Predicted values were obtained from sensitivity analyses applying the primary modelling specification to individual epigenetic clocks; the primary analysis used a unitless composite measure based on the three clocks. See Supplementary Materials for details.
