# Supplementary material for: Residential exposure to deadly gun violence and accelerated biological aging in a national sample of U.S. adolescents
Source: SSM Popul Health. 2026 Jun 19;35:101937. doi: 10.1016/j.ssmph.2026.101937 (PMC13316161; doi:10.1016/j.ssmph.2026.101937)
Supplement: Multimedia component 1 [file mmc1.docx]

**Supplemental Materials**

Residential Exposure to Deadly Gun Violence and Accelerated Biological Aging in a National Sample of Adolescents

**Supplemental Methods**

DNA Methylation Processing pg. 2

Epigenetic clocks pg. 2

Biological aging summary scores pg. 3

Gun Violence Archive pg. 3

Exposure measures pg. 4

Propensity score matching pg. 4

Weighted regression analyses pg. 5

**Supplemental Results**

Effect size interpretations pg. 5

**Supplemental Tables**

Table S1: Factor analyses for biological aging summary scores in matched sample pg. 6

Table S2: Characteristics of the full analytic cohort pg. 7

Table S3: Logistic regression odds ratios from propensity score matching pg. 8

Table S4: Estimated associations in the overall and matched samples pg. 9

**Supplemental Figures**

Figure S1: Std. mean differences in matching parameters pg. 10

Figure S2: Distribution of propensity scores among treated and control groups pg. 11

Figure S3: Associations for levels of exposure intensity in the matched sample pg. 12

Figure S4: Associations for distance-specific exposure counts in the matched sample pg. 13

Figure S5: Estimates for individual epigenetic clocks in overall and matched samples pg. 14

**Supplemental References** pg. 15

Supplemental Methods

*DNA Methylation Collection and Processing*

To obtain and process DNAm data, saliva samples were provided by participants at Year 9 and Year 15 follow-up waves [1]. Saliva samples were collected by contracted FFCWS staff during in-home interviews at Year 9 and Year 15 using the Oragene DNA Self-Collection Kit. For those who did not complete a home interview at Year 15 (all participants completed home interviews at Year 9), saliva collection kits were sent to participants via mail and after collection participants shipped the kits to contracted FFCWS staff. Specimen containers were then shipped to Princeton University for DNAm processing.

DNA extraction followed the Oragene prepIT•L2P Laboratory Protocol for manual DNA purification. Extracted DNA concentration was subjected to bisulfite conversion and analyzed with the Illumina Infinium Human Methylation450K (450K) or Illumina Infinium MethylationEPIC (EPIC) array according to the manufacturer’s protocol. Year 9 and Year 15 samples were run at the same time to minimize technical variation. Quality control procedures removed probes with detection values greater than 0.01 or 0.05 for the 450K or EPIC arrays, respectively, and those with methylated or unmethylated bead counts of less than four. Samples were removed if they had outlier methylation or bisulfite conversion values, or if the sex predicted from the methylation data differed from the recorded sex. As an additional safeguard against residual platform effects, all analytic models adjusted for array chip (EPIC vs. 450K) as a covariate.

*Epigenetic Clocks*

Epigenetic clock values were obtained directly from the FFCWS Biomarker data release, which computed each clock using algorithms and procedures specified by its developers [1]. Because subsets of clock probes are unavailable on one of both array platforms, the released algorithms address differences between the 450K and EPIC arrays by imputing missing probes against clock-specific reference standards where the original developers prescribe doing so. For GrimAge and PhenoAge, we used the principal components (PC) versions of each clock, which were developed specifically to reduce technical noise, including platform- and batch-related variation, and to improve test-retest reliability [3]. Clock-specific estimates from regression analyses in the overall and matched samples are presented in Figure S5.

GrimAge was developed to estimate mortality risk using data from 2,356 individuals in the Framingham Heart Study [4]. Separate blood-based DNAm profiles of twelve plasma proteins and smoking pack years were identified then regressed on time-to-death due to all-cause mortality using an elastic net Cox regression model, adjusting for chronological age and sex. Raw values of the selected covariates were then transformed to be in the unit of years to generate a final measure of GrimAge, which demonstrated high predictive ability of lifespan and time-to-coronary heart disease in validation analyses [4].

PhenoAge was developed as a lifespan predictor using data from 9,926 individuals in the National Health and Nutrition Examination Survey (NHANES) III [5]. In the first step, an individual’s phenotypic age was estimated by regressing the mortality hazard on a set of nine clinical aging-related biomarkers and chronological age using a Cox penalized regression model. Next, values of PhenoAge were estimated using elastic net regression to identify specific regions of blood-based DNAm that were associated with phenotypic age. Validation analyses indicated that PhenoAge is highly predictive of a variety of aging outcomes, including all-cause mortality, cancers, and physical functioning across tissue and cell types [5].

DunedinPACE was developed to predict an individual’s pace of aging using longitudinal data from 1,037 participants in the Dunedin Study [6]. Researchers used elastic net regression to identify specific regions of DNAm that were associated with physiological changes over a 20-year period for 19 biomarkers of organ system integrity, physical function, and cognitive performance. The resulting measure of DunedinPACE was shown in validation studies to be highly predictive of incident morbidity, disability, and mortality [6].

*Summary Scores*

We calculated summary scores for the primary outcome, Y15 biological aging, using standardized age acceleration values for GrimAge PC, PhenoAge PC, and DunedinPACE. We standardized summary scores because the metrics for interpreting estimates from DunedinPACE (e.g., proportional change in biological aging per year of chronological age) differ from GrimAge and PhenoAge (e.g., the deviation between biological age and chronological age). We created a similar summary score at Y9 to account for pre-exposure differences in biological aging in regression models. Summary scores were calculated separately for analyses using the full analytic cohort, the matched sample, and sensitivity analyses with restricted sample.

We tested longitudinal measurement invariance across the two assessment waves for the matched sample using weighted robust maximum likelihood. The configural model demonstrated good fit (CFI = 0.995, RMSEA = 0.077, SRMR = 0.045). Although the robust chi-square difference tests for metric invariance was statistically significant (Δχ^2^(3) = 9.85, p = 0.020), changes in practical fit indices were negligible (ΔCFI = -0.004, ΔRMSEA = 0.003), supporting practical measurement invariance. Scalar invariance was supported (Δχ^2^(2) = 3.60, p = 0.166; ΔCFI = -0.001). Taken together, these findings indicate that the biological aging summary score construct was measured equivalently over time, permitting meaningful comparisons of latent means and change.

*Gun Violence Archive*

The Gun Violence Archive is a unique national data source for epidemiological research on community gun violence. Traditionally used national and state data sources on firearm violence and injury (e.g., National Violent Death Reporting System, FBI Uniform Crime Reporting Program) often aggregate data at the city or county level, precluding researchers from using those data to study local-level counts of community gun violence. In contrast, publicly available data from the GVA includes granular information on the specific time and location of shootings, similar to that found in police department data. The GVA has been shown to be a valid data source for community firearm violence, with an overall sensitivity of 81% and positive predictive value of 99% compared to police department databases, and strong correlations with aggregate data from the Center for Disease Control and Prevention mortality records [7,8]. Additional details on the methodology of the GVA and comparisons with CDC reports of gun deaths are located in FFCWS documentation [8].

*Exposure Measures*

Data from the Gun Violence Archive linked to the FFCWS includes incident counts of fatal shootings in the prior year at 100-meter distance intervalsfrom participants’ homes at Y15. Beyond the primary continuous 1,600m count described in the main text, we constructed three alternative operationalizations for sensitivity analyses: (1) a dichotomous any-versus-none indicator; (2) a 4-level categorical measure of exposure intensity (unexposed: 0 incidents, low: 1, moderate: 2-4, and high: 5+); 3); and (3) continuous counts at 1,000m and 500m.

*Propensity Score Matching*

We estimated adolescents’ propensity to reside in neighborhoods affect by deadly gun violence based on the following covariates: self-identified race/ethnicity, tract-level percent poverty (Y9 & Y15), maternal income-poverty ratio (Y9 & Y15), maternal educational attainment (Y15), and county-level violent crime rates (Y9). Matching parameters included time-varying Y9 measures to account for baseline characteristics that may influence selection into neighborhoods with higher levels of gun violence. Due to the unavailability of GVA data at Y9, we used county-level violent crime rates as a prior indicator of exposure to deadly gun violence, though at a broader scale than Y15 GVA data. We omitted Y9 maternal educational attainment as a matching parameter due to collinearity with Y15 maternal educational attainment.

First, we estimated matching weights for propensity to any gun violence exposure conditional on the matching covariates among participants in the full analytic cohort. Using a generalized linear model with a logit link function, we specified a full optimal matching method, which pairs each treated unit with one or more control units (and vice versa), assigning weights to optimize covariate balance. This approach maximizes the use of available data by ensuring all units are retained in the analysis. We implemented exact matching on self-identified race/ethnicity and applied a 0.25 SD caliper for socioeconomic covariates. Given the large variance within the sample, Y9 violent crime rates were matched using a 1.0 SD caliper. Estimated odds ratios for propensity to deadly gun violence exposure are reported in Table S2.

The resulting matched sample included 499 adolescents with 265 treatment units (i.e., at least 1 incident of deadly gun violence within 1600m of home) and 234 (weighted n=153.61) control units (i.e., no incidents within 1600m of home). The weighted N (effective sample size; ESS) for the matched control group was computed using the Kish formula: ESS = (Σwᵢ)² / Σwᵢ², where wᵢ are the matching weights assigned to control units by the full matching procedure [9]. This formula accounts for the unequal weighting of matched controls and reflects the loss of precision relative to an equally-weighted sample of the same size. We assessed covariate balance in the treated and control groups by comparing standardized mean differences (threshold: 0.1) across adjusted and unadjusted models (Figure S1). Next, we inspected weighted descriptive statistics for the matched sample (Table 1) and overlap in the distribution of propensity scores among matched treated and control units and unmatched treated and control units (Figure S2).

*Weighted Regression Analyses*

We then conducted weighted regression analyses in the matched sample to estimate the ATT and cluster-robust 95% confidence intervals. Before conducting these analyses, we estimated and standardized biological aging summary scores within the matched sample. Given that standardized mean differences in matching covariates were less than 0.10 within the matched sample (Figure S2), it was no longer necessary to condition the ATT on these covariates [10]. Weighted regression models adjusted for non-matching covariates specified in the main text, with Y9 and Y15 values included for chronological age and cell-type estimates.

**Supplemental Results**

*Effect Size Interpretation*

We estimated that a 15-year-old living in an urban neighborhood with average levels of deadly gun violence is biologically about 2.5 months older or aging 5.3% faster than expected had they lived in a neighborhood without fatal shootings. These interpretations were derived from unstandardized estimates for GrimAge, PhenoAge, and DunedinPACE in weighted linear regression models among the matched sample. The multiple unit interpretations reflect differences in units across clocks: GrimAge and PhenoAge reflect years of accelerated or decelerated biological aging, whereas DunedinPACE reflects a rate of biological aging relative to chronological time, expressed as a percent increase or decrease.

The 2.5-month difference in biological aging between exposed participants and their counterfactual unexposed scenario was estimated as the mean predicted values for GrimAge (2.2 months) and PhenoAge (2.8 months) at the average level of exposure (3.24 incidents) among the treated group (Table 1). Alternatively, adolescents’ pace of biological aging increased 5.3%, as indicated by predicted values for DunedinPACE at average levels of exposure. Predicted values for the unitless summary score measure corresponded to a 0.031 standard deviation increase in biological aging.

**Supplementary Tables**

| Table S1. Factor loadings for biological aging summary scores from confirmatory factor analyses in the matched sample (n=499). | | | | | | | |
| --- | --- | --- | --- | --- | --- | --- | --- |
| Outcome | Std. Factor Loading | Standard Error | z-value | p-value | Residual Variance | AIC | BIC |
| Biological Aging Y15 |  |  |  |  |  | 3300.28 | 3325.55 |
| Y15 PC GrimAge | 0.833 | 0.050 | 17.103 | <0.001 | 0.306 |  |  |
| Y15 PC PhenoAge | 0.980 | 0.049 | 21.774 | <0.001 | 0.040 |  |  |
| Y15 DunedinPACE | 0.838 | 0.036 | 24.362 | <0.001 | 0.298 |  |  |
| Biological Aging Y9 |  |  |  |  |  | 3342.10 | 3367.38 |
| Y9 PC GrimAge | 0.789 | 0.047 | 16.520 | <0.001 | 0.364 |  |  |
| Y9 PC PhenoAge | 0.945 | 0.045 | 20.987 | <0.001 | 0.108 |  |  |
| Y9 DunedinPACE | 0.839 | 0.037 | 23.431 | <0.001 | 0.296 |  |  |
| Note: PC GrimAge and PC PhenoAge were residualized for chronological age; PC versions are not available for DunedinPACE. Comprehensive fit statistics are not presented because models are just-identified with zero degrees of freedom. Scalar measurement invariance was demonstrated across both time points (Δχ^2^(2) = 3.60, p = 0.166; ΔCFI = -0.001). Factors loadings and tests of measurement invariance were nearly identical to those in the overall sample (n=1,781). | | | | | | | |

| **Table S2.** Characteristics of the full analytic cohort of adolescents from the Future of Families and Child Well-being Study (n=1,781) | | | | | |  |
| --- | --- | --- | --- | --- | --- | --- |
| Year 15 Variable | Overall  (N=1,781) | Black  (n=820) | Hispanic  (n=465) | White  (n=339) | Other/Multiple Race  (n=157) | |
| Chronological age, mean (SD), years | 15.53 (0.63) | 15.59 (0.65) | 15.55 (0.69) | 15.42 (0.53) | 15.40 (0.53) | |
| Sex, n (%) |  |  |  |  |  | |
| Female | 879 (49.35) | 413 (50.37) | 222 (47.74) | 169 (49.85) | 75 (47.77) | |
| Male | 902 (50.65) | 407 (49.63) | 243 (52.26) | 170 (50.15) | 82 (52.23) | |
| Maternal educational attainment, n (%) |  |  |  |  |  | |
| Less than high school | 310 (17.41) | 125 (15.24) | 144 (30.97) | 25 (7.37) | 16 (10.19) | |
| High school | 324 (18.19) | 148 (18.05) | 99 (21.29) | 55 (16.22) | 22 (14.01) | |
| Some college | 783 (43.96) | 427 (52.07) | 168 (36.13) | 119 (35.10) | 69 (43.95) | |
| College | 364 (20.44) | 120 (14.63) | 54 (11.61) | 140 (41.30) | 50 (31.85) | |
| Maternal income-to-poverty ratio, mean (SD) | 2.42 (2.63) | 1.70 (1.57) | 2.15 (2.09) | 4.24 (3.81) | 3.06 (3.32) | |
| Census tract poverty, mean (SD) | 21.74 (14.72) | 27.49 (14.93) | 20.35 (12.61) | 11.75 (10.08) | 17.40 (13.98) | |
| Exposure to deadly gun violence, n (%) |  |  |  |  |  | |
| None | 867 (48.68) | 278 (32.90) | 232 (40.89) | 269 (79.35) | 88 (56.05) | |
| At least one | 914 (51.32) | 542 (66.10) | 233 (50.11) | 70 (20.65) | 69 (43.95) | |
| Exposure count, mean (SD) | 2.38 (4.48) | 3.64 (5.52) | 1.78 (3.25) | 0.36 (0.88) | 1.96 (4.09) | |
|  | | | | | |  |

| Table S3. Logistic regression odds ratios from propensity score matching (n=1,781). | |  |
| --- | --- | --- |
| Covariate | OR (95% CI) | |
| Intercept | 0.27 (0.17, 0.42) | |
| Self-identified race/ethnicity (ref: Black) |  | |
| White | 0.45 (0.31, 0.64) | |
| Hispanic | 0.93 (0.70, 1.24) | |
| Multi-racial/some other race | 0.85 (0.56, 1.29) | |
| Y15 Maternal income-poverty ratio | 1.01 (0.95, 1.09) | |
| Y15 Maternal education (ref: <HS) |  | |
| HS | 0.59 (0.40, 0.86) | |
| Some college | 0.93 (0.67, 1.30) | |
| College | 0.67 (0.44, 1.03) | |
| Y15 Tract percent poverty | 1.07 (1.06, 1.09) | |
| Y9 Maternal income-poverty ratio | 1.00 (0.92, 1.08) | |
| Y9 Tract percent poverty | 1.02 (1.00, 1.03) | |
| Y9 County violent crime rate | 1.40 (1.23, 1.59) | |
| Notes: The outcome was any prior-year exposure to deadly gun violence within 1600m of home at Y15. Full matching was performed exactly on race/ethnicity and standardized calipers for socioeconomic covariates (see Supplemental Methods). Y9 maternal educational attainment was omitted due to collinearity with Y15 educational attainment. | |  |

| Table S4. Estimated associations between exposure to deadly gun violence and biological aging in the overall and matched samples | | | | | | |  |
| --- | --- | --- | --- | --- | --- | --- | --- |
| Exposure Specification | Overall Sample (N=1,781) | | | | | Matched Sample (n=499) | |
|  | Model 1 | Model 2 | Model 3 | Model 4 | Model 5 | Model 6 | |
|  | β (95% CI) | β (95% CI) | β (95% CI) | β (95% CI) | β (95% CI) | β (95% CI) | |
| Primary: Exposure Count 1,600m | 0.060 (0.037, 0.083) | 0.049 (0.025, 0.072) | 0.025 (-0.000, 0.050) | 0.006 (-0.018, 0.031) | 0.005 (-0.011, 0.020) | 0.033 (0.003, 0.064) | |
| Sensitivity Analyses |  |  |  |  |  |  | |
| Any-vs-None Exposure (1,600m) | 0.150 (0.110, 0.190) | 0.126 (0.085, 0.167) | 0.083 (0.035, 0.130) | 0.042 (-0.003, 0.088) | 0.018 (-0.013, 0.050) | 0.042 (-0.025, 0.108) | |
| Exposure Intensity (ref: none) |  |  |  |  |  |  | |
| Low (1 incident; N=300, n=116) | 0.122 (0.066, 0.179) | 0.105 (0.048, 0.162) | 0.084 (0.025, 0.143) | 0.059 (0.002, 0.115) | 0.027 (-0.011, 0.066) | 0.025 (-0.052, 0.102) | |
| Moderate (2-4 incidents; N=361, n=104) | 0.147 (0.095, 0.199) | 0.121 (0.068, 0.174) | 0.073 (0.016, 0.132) | 0.033 (-0.023, 0.088) | 0.021 (-0.018, 0.059) | 0.046 (-0.030, 0.121) | |
| High (5+ incidents; N=253, n=45) | 0.190 (0.134, 0.246) | 0.161 (0.103, 0.218) | 0.097 (0.030, 0.164) | 0.022 (-0.044, 0.088) | -0.010 (-0.058, 0.037) | 0.074 (-0.035, 0.183) | |
| Exposure Spatial Sensitivity |  |  |  |  |  |  | |
| Exposure Count 1,600m (unstd) | 0.013 (0.008, 0.018) | 0.011 (0.005, 0.016) | 0.005 (0.000, 0.011) | 0.001 (-0.004, 0.007) | 0.001 (-0.002, 0.004) | 0.009 (0.001, 0.018) | |
| Exposure Count 1,000m (unstd) | 0.024 (0.014, 0.034) | 0.020 (0.009, 0.030) | 0.010 (-0.001, 0.020) | 0.003 (-0.007, 0.014) | 0.001 (-0.005, 0.008) | 0.018 (-0.001, 0.038) | |
| Exposure Count 500m (unstd) | 0.054 (0.028, 0.080) | 0.043 (0.016, 0.069) | 0.022 (-0.004, 0.049) | 0.010 (-0.016, 0.036) | 0.005 (-0.014, 0.024) | 0.025 (-0.017, 0.066) | |
| Exposure Count 1,600m |  |  |  |  |  |  | |
| Restrict Sample to 1yr Exposure Data (N=1,350; n=284) | 0.059 (0.034, 0.084) | 0.049 (0.024, 0.074) | 0.027 (0.000, 0.053) | 0.010 (-0.016, 0.037) | 0.003 (-0.013, 0.020) | 0.051 (0.015, 0.088) | |
| Control for Y15 County Violent Crime | 0.045 (0.021, 0.069) | 0.039 (0.014, 0.063) | 0.021 (-0.004, 0.046) | 0.009 (-0.016, 0.034) | 0.007 (-0.009, 0.022) | 0.038 (0.008, 0.068) | |
| Match on Y15 County Violent Crime (n=379) | -- | -- | -- | -- | -- | 0.039 (0.004, 0.075) | |
| Note: All exposure measures examined in separate models within the overall and matched samples. Continuous exposure measures were standardized within each sample. Linear regression was used in the overall sample and weighted linear regression was used in the matched sample. All analyses calculated 95% confidence intervals using cluster-robust standard errors. | | | | | | |  |

**Supplemental Figures**

**
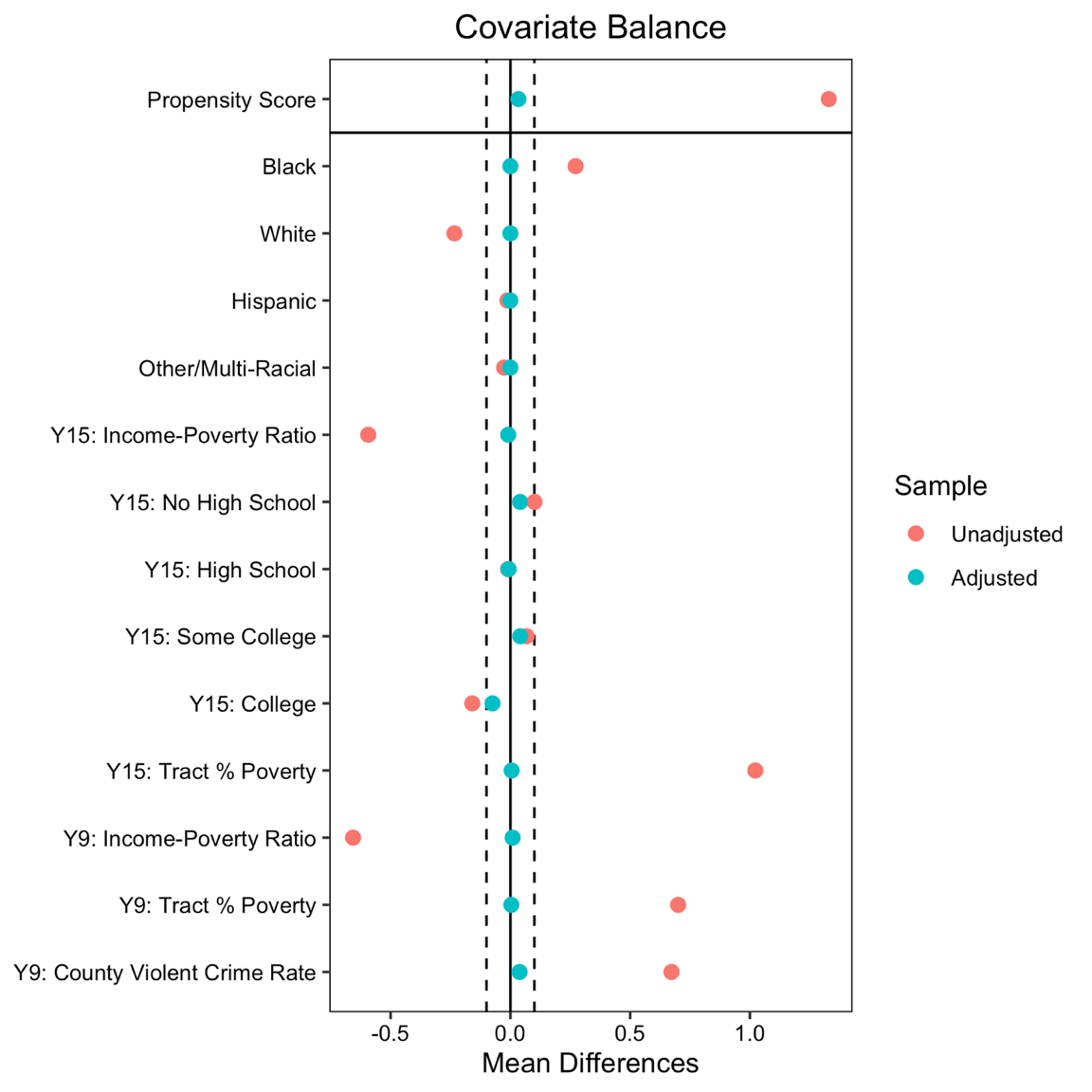
Figure S1.** Standardized mean differences in matching parameters (n=499)

Caption: Estimates calculated using full optimal matching with exact matching on self-identified race/ethnicity and caliper matching on socioeconomic covariates and violent crime rates. Dotted lines indicate the 0.10 threshold for standardized mean differences.

**
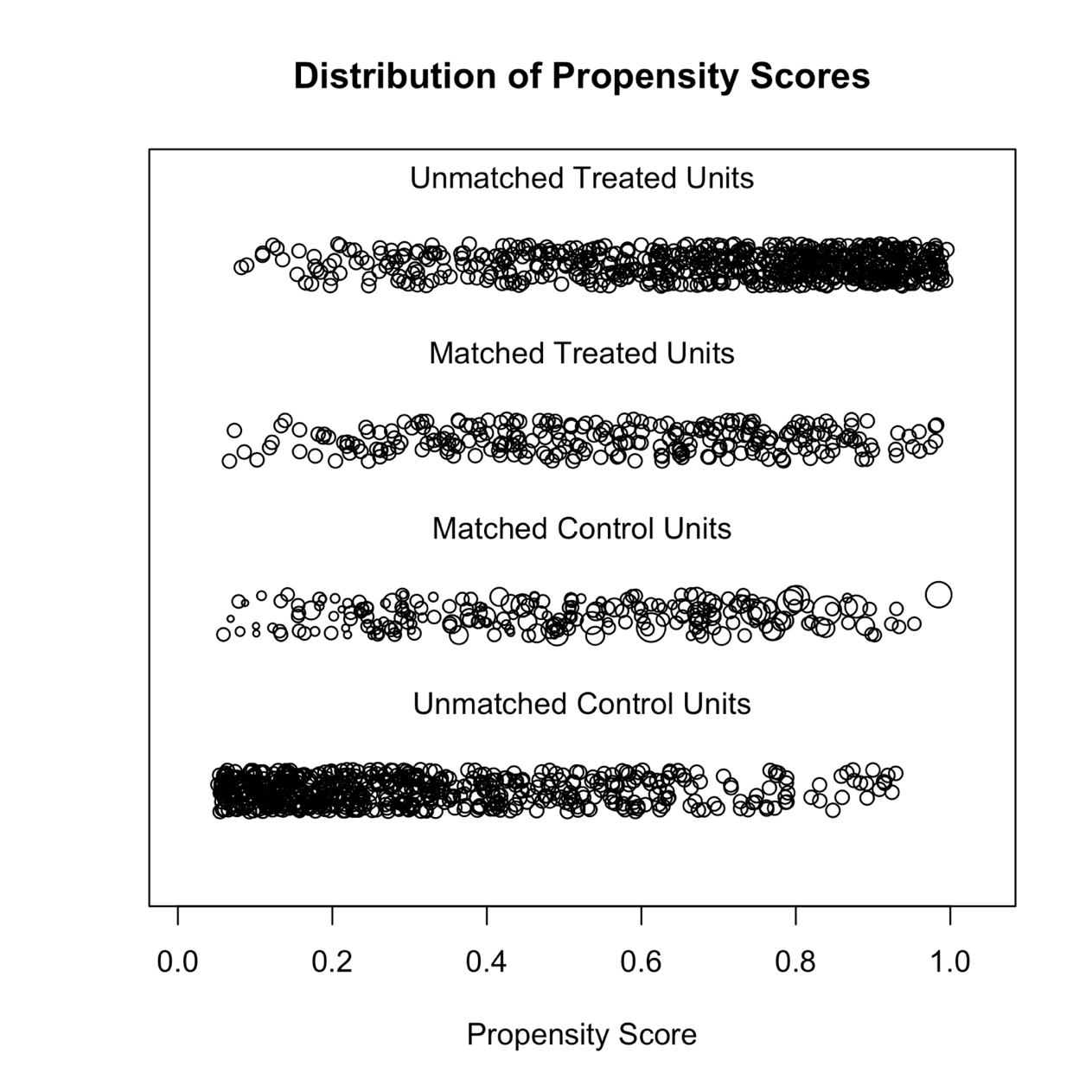
Figure S2.** Distribution of propensity scores among treated control groups

Caption: N matched = 499 (n treated = 265, n control = 234 [weighted n = 153.61]); N unmatched = 1282 (n treated = 649, n control = 633).

**Figure S3: Estimates from sensitivity analyses examining associations between levels of exposure intensity and biological age acceleration in the matched sample (n=499)**


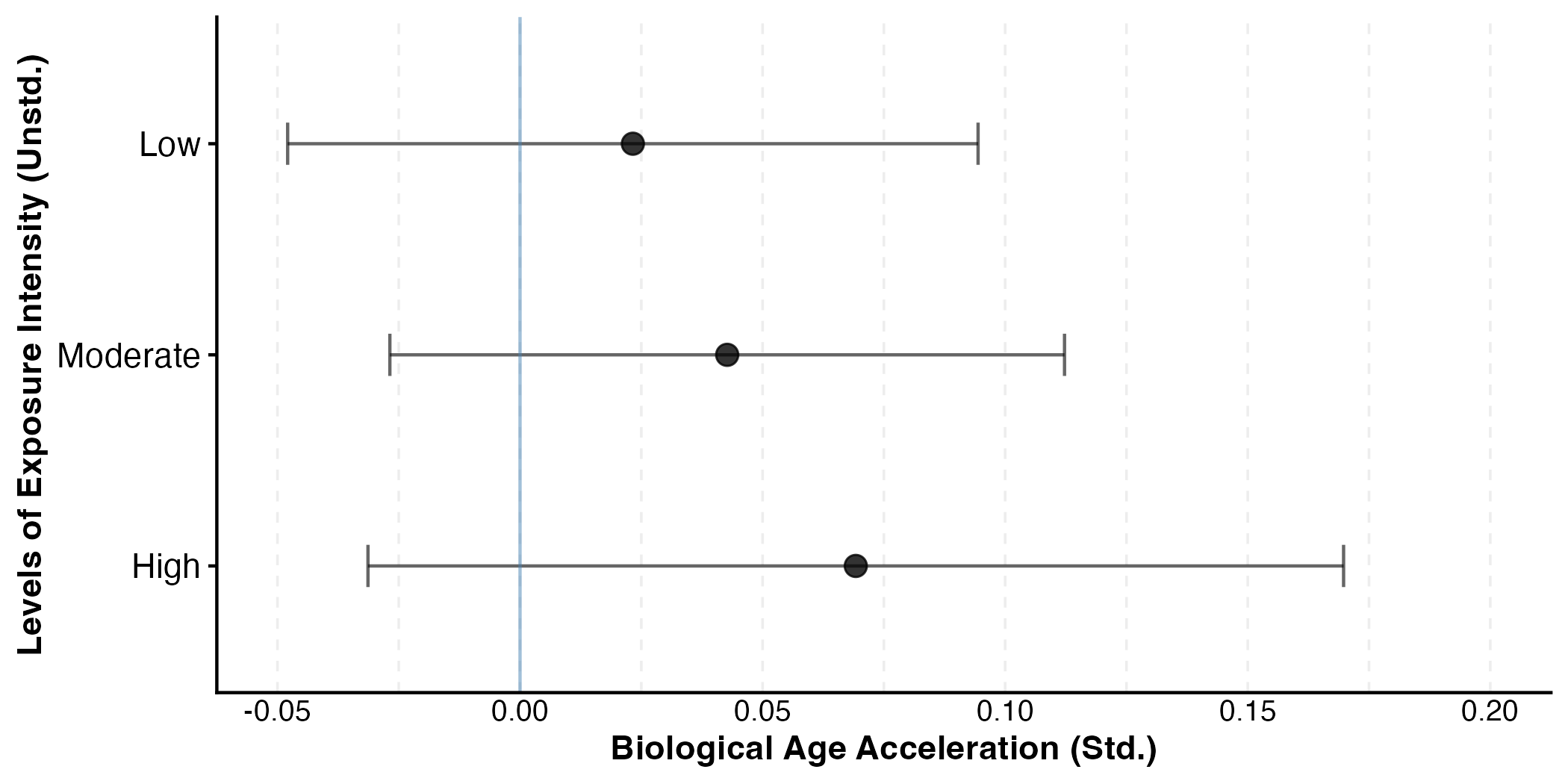


Caption: Results of sensitivity analyses examining associations between exposure intensity (ref: unexposed) and biological age acceleration among adolescents in the matched sample. Estimates targeting the average treatment effect on the treated (ATT) were calculated using weighted regression models in the matched sample (n=499). Exposure intensity was measured as a categorical variable of incident counts, where Low = 1 prior-year incident within 1600 of home (n = 116), Moderate = 2-4 incidents (n = 104), and High=5 or more incidents (n = 45). Wide confidence intervals for ATT estimates reflect the relatively lower variance in exposure intensity categories.

**Figure S4. Estimates from sensitivity analyses examining associations between distance-specific exposure counts and biological age acceleration in the matched sample (n=499)**


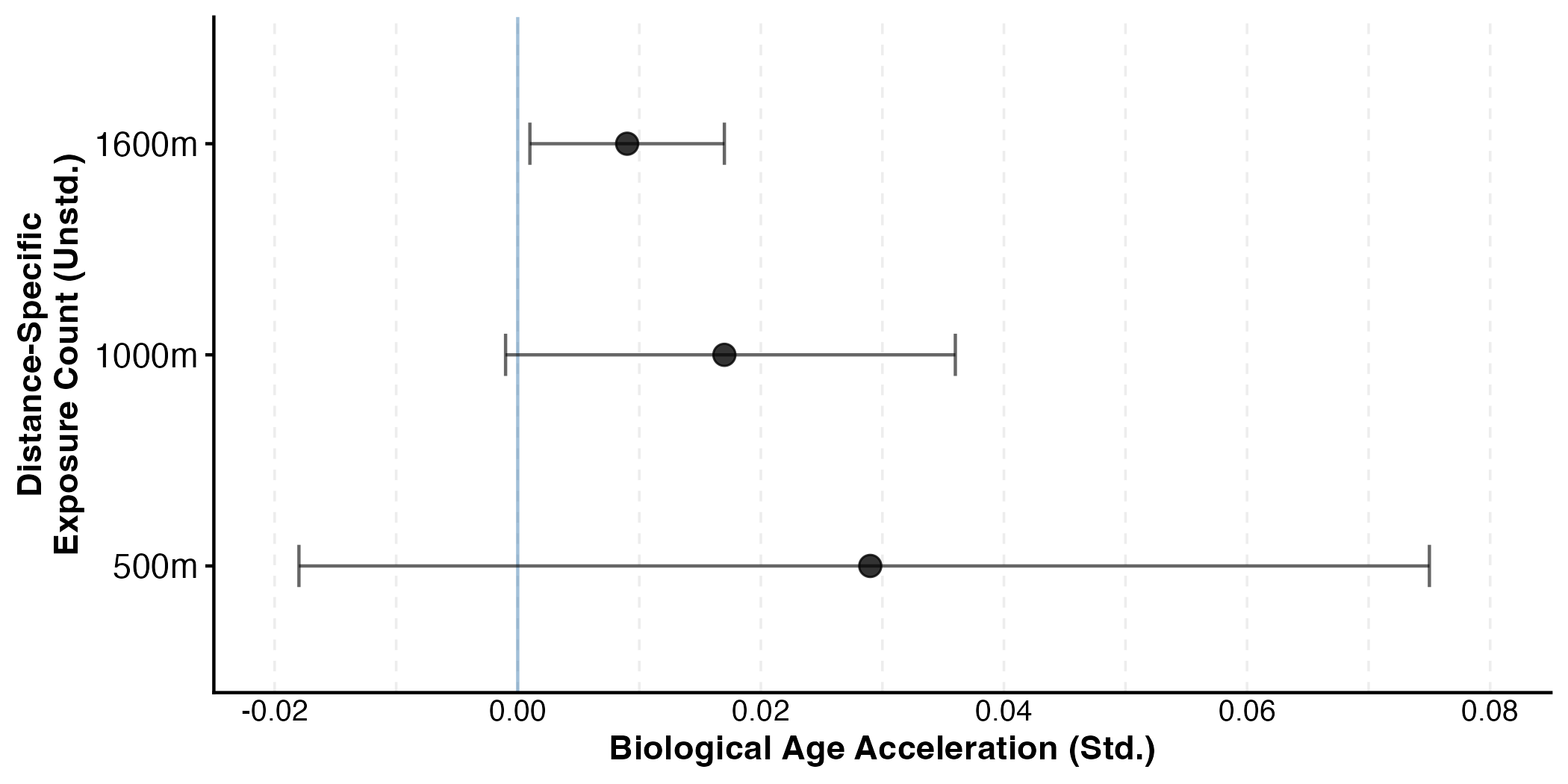


Caption: Results of sensitivity analyses examining associations between the count of deadly incidents within 1600m, 1000m, and 500m of home in the past year and biological age acceleration among adolescents in matched sample. Estimates targeting the average treatment effect on the treated (ATT) were calculated using weighted regression models in the matched sample (n=499). Widening confidence intervals at closer proximities reflect lower exposure variance in the matched sample (n=499, 17% exposed to any incidents ≤ 500m, range: 0-4).

**Figure S5: Estimates from sensitivity analyses for comparing individual epigenetic clocks to the biological aging summary score measure in the overall (n=1,781) and matched (n=499) samples**

**
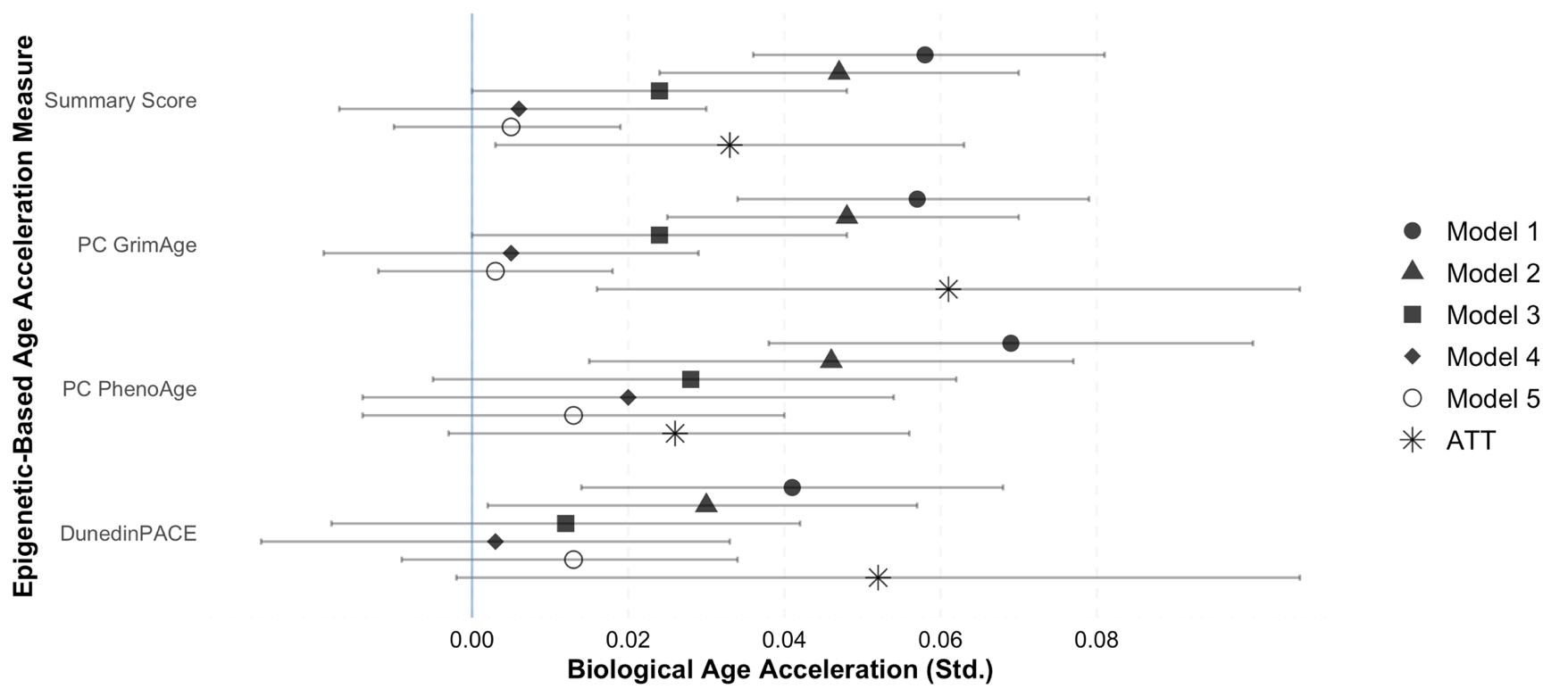
**

Caption: Results from sensitivity analyses that examined associations between incident counts within 1600m of home and each epigenetic clock used to create the biological aging summary score (plotted for comparison). Model 1 adjusted for Y15 covariates: chronological age, sex, maternal prenatal smoking, array chip, and cell type estimates. Model 2 further adjusted for maternal income-to-poverty ratio and maternal educational attainment. Model 3 for census tract percent poverty, Model 4 for self-identified race/ethnicity, and Model 5 for time-varying Y9 covariates, including biological aging, cell type estimates, chronological age, maternal income-to-poverty ratio, census tract percent poverty, as well as Y9 county-level violent crime rates. Among the matched sample, models estimated the average treatment effect on the treated (ATT) using fully adjusted weighted regression analyses (n treatment = 265, n control = 234).

**Supplemental References**

1. Bendheim-Thoman Center for Research on Child Wellbeing, Columbia Population Research Center, Department of Molecular Biology, Princeton University, Population, Neurodevelopment, and Genetics Program, University of Michigan. *The Future of Families and Child Wellbeing Study Biomarker Appendage: 9- and 15-Year Follow-Up Waves*. 2023. Accessed March 13, 2023. https://ffcws.princeton.edu/sites/g/files/toruqf4356/files/documents/Biomarker_Documentation_2023.03.10.pdf

2. Horvath S. DNA methylation age of human tissues and cell types. *Genome Biology*. 2013;14(10):3156. doi:10.1186/gb-2013-14-10-r115

3. Higgins-Chen AT, Thrush KL, Wang Y, et al. A computational solution for bolstering reliability of epigenetic clocks: implications for clinical trials and longitudinal tracking. *Nat Aging*. 2022;2(7):7. doi:10.1038/s43587-022-00248-2

4. Lu AT, Quach A, Wilson JG, et al. DNA methylation GrimAge strongly predicts lifespan and healthspan. *Aging*. 2019;11(2):303-327. doi:10.18632/aging.101684

5. Levine ME, Lu AT, Quach A, et al. An epigenetic biomarker of aging for lifespan and healthspan. *Aging (Albany NY)*. 2018;10(4):573-591. doi:10.18632/aging.101414

6. Belsky DW, Caspi A, Corcoran DL, et al. DunedinPACE, a DNA methylation biomarker of the pace of aging. Deelen J, Tyler JK, Suderman M, Deelen J, eds. *eLife*. 2022;11:e73420. doi:10.7554/eLife.73420

7. Gobaud AN, Mehranbod CA, Kaufman E, et al. Assessing the Gun Violence Archive as an Epidemiologic Data Source for Community Firearm Violence in 4 US Cities. *JAMA Network Open*. 2023;6(6):e2316545. doi:10.1001/jamanetworkopen.2023.16545

8. Bendheim-Thoman Center for Research on Child Wellbeing, Columbia Population Research Center. *Fragile Families Gun Violence Archive Data on Local Deadly Gun Violence Restricted Use Appendage: Year 15 Follow-Up Wave*. 2019. Accessed September 13, 2024. https://ffcws.princeton.edu/sites/g/files/toruqf4356/files/documents/ff_gva_15y_res1_20190603.pdf

9. Kish L. Sampling Organizations and Groups of Unequal Sizes. *American Sociological Review*. 1965;30(4):564. doi:10.2307/2091346

10. Nguyen TL, Collins GS, Spence J, et al. Double-adjustment in propensity score matching analysis: choosing a threshold for considering residual imbalance. *BMC Med Res Methodol*. 2017;17(1):78. doi:10.1186/s12874-017-0338-0
